# ﻿The polyphasic approach reveals two new species and two new records of *Nigrospora* (Apiosporaceae, Amphisphaeriales) associated with *Aquilaria sinensis* from China

**DOI:** 10.3897/mycokeys.121.154055

**Published:** 2025-08-21

**Authors:** Shiyu Zhang, Junfu Li, Hongbo Jiang, Shuang Ye, Ausana Mapook, Jianchu Xu, Kevin D. Hyde, Prapassorn Damrongkool Eungwanichayapant

**Affiliations:** 1 School of Science, Mae Fah Luang University, Chiang Rai 57100, Thailand; 2 Center for Mountain Futures, Kunming Institute of Botany, Kunming 650201, China; 3 CIFOR-ICRAF China Program, World Agroforestry (ICRAF), Kunming 650201, China; 4 Center of Excellence in Fungal Research, Mae Fah Luang University, Chiang Rai 57100, Thailand; 5 Tea Research Institute, Yunnan Academy of Agricultural Sciences, Kunming 650201, China

**Keywords:** 2 new species, asexual fungi, endophyte, Hyphomycetes, multigene phylogeny, Sordariomycetes

## Abstract

This study isolated endophytic fungi of the genus *Nigrospora* from *Aquilaria sinensis* (Chinese agarwood) in Guangxi, China. Through rigorous morphological comparisons and molecular phylogenetic analyses, we have identified two new species (*N. guangxiensis* and *N. pubeiensis*) and two new host-recorded species (*N. oryzae* and *N. camelliae-sinensis*). The morphological analysis revealed that the new species align with the genus-level definition of *Nigrospora* in colony morphology, conidiophores, and conidial characteristics. However, they were distinguished from known species by finer features such as conidial dimensions and conidiogenous structures. For instance, conidiophores in *Nigrospora* are typically differentiated from hyphae, characterized by dark pigmentation, which is consistent with broader taxonomic descriptions. Molecular phylogeny, based on concatenated datasets of the ITS, *TEF1-α*, and *TUB2* loci, showed that *N. pubeiensis* is most closely related to *N. chinensis*, while *N. guangxiensis* forms a distinct basal clade relative to these two groups. Additionally, *N. oryzae* and *N. camelliae-sinensis* were first recorded on *A. sinensis*, expanding their known host ranges. The study enriches the taxonomic framework of *Nigrospora* by providing novel data from China, emphasizing the importance of integrating morphological and molecular approaches in fungal systematics. It also underscores the ecological diversity of fungal-host interactions in agarwood-producing trees, a topic explored in related research on endophytic fungi. These findings have significant implications for our understanding of fungal diversity and their ecological roles in agarwood-producing trees, and they pave the way for further research in this area.

## ﻿Introduction

Species of *Nigrospora* Zimm. are pathogens, endophytes, or saprobes on various plant hosts in different habitats (Wang et al. 2017). This genus belongs to Apiosporaceae K.D. Hyde, J. Fröhl., Joanne E. Taylor & M.E. Barr (Hyde et al. 2024). *Nigrospora* was introduced by Zimmerman (1902) for *N. panici* Zimm., isolated from the dead leaves of *Panicum amphibium* Steud, in Indonesia. It was known from the asexual characters because of its black opaque conidial spores borne from grey conidiophores. Mason (1927) classified four species occurring on monocotyledons, *viz.*, *N. oryzae* (Berk. & Broome) Petch, *N. arundinacea* (Cooke & Massee) Potl., *N. sacchari* (Speg.) E.W. Mason, and *N. sphaerica* (Sacc.) E.W. Mason, by their black-spored hyphomycetous characters and further indicated *N. javanica* Palm as a synonym of *N. panici*. Species of *Nigrospora* were delimited based on morphological characteristics until it was found that some key morphological characteristics overlapped between phylogenetically distinct species, such as conidial dimensions (Kirk et al. 2008). To address this issue, Wang et al. (2017) analyzed recent species in the genus *Nigrospora* and introduced twelve novel species based on morphological characters, host associations, and a multigene combined phylogeny of ITS, *TEF1-α*, and *TUB2*. They further placed this genus within the Apiosporaceae (Xylariales). Moreover, Hao et al. (2020) reported *N. gorlenkoana* Novobr., *N. oryzae*, *N. osmanthi* Mei Wang & L. Cai, *N. rubi* Mei Wang & L. Cai, and *N. sphaerica* with 13 novel host associations, including economically important crops from China. They confirmed the genus in the family Apiosporaceae based on multi-gene phylogenetic analyses. Liu et al. (2024) isolated *N. anhuiensis* Y. Liu & F. He as a pathogen from rice in Anhui Province, China. Zou et al. (2024) established a novel species, *N. yunnanensis* M.T. Zou & Yong Wang, from *Juglans regia* L. in Yunnan Province, China.

The secondary metabolites of *Nigrospora* species typically exhibit good biological activities and are thus regarded as an interesting source of natural products due to their potential application value. For example, *Nigrospora sphaerica* produces hydroanthraquinones, which exhibit anti-inflammatory activity (Kuang et al. 2022). The metabolites of this species have also shown prominent herbicidal activity in the treatment of intact, greenhouse-grown plants (Fukushima et al. 1998). *Nigrospora sphaerica* and *Nigrospora camelliae-sinensis* Mei Wang & L. Cai have also been proven to be a rich source of other secondary metabolites, such as hydroanthraquinone diketopiperazines, lactones, isochromene, and cyclopentanone derivatives (Metwaly et al. 2014; Zhang et al. 2015; Wu et al. 2018; Zhu et al. 2018; Huang et al. 2021; Kuang et al. 2022). Some have been used in the production of green tea, textile dyes, and food preservatives (Xu et al. 2022).

*Aquilaria sinensis* (Lour.) Spreng. (Thymelaeaceae) is an important economic and medicinal material in China. The high-value, fragrant, and dark resinous heartwood of *A. sinensis* is known by its popular name “agarwood” (Liu et al. 2017; Wang et al. 2018; Tan et al. 2019; Wang et al. 2019). The damage and infection of the white wood of *A. sinensis* stimulate the plant’s defense response, and subsequently, dark resin is produced to suppress further infection, resulting in the formation of agarwood (Chen et al. 2017; Chhipa et al. 2017; Faizal et al. 2020). Fungal inoculation is considered to be effective in the agarwood-producing industry, and most of the fungi used for inoculation are endophytes from agarwood-producing trees, including *A. sinensis* (Han et al. 2014; Azren et al. 2018; Subasinghe et al.2018; Du et al. 2024). Du et al. (2022b) reviewed the biological activity and agarwood induction potential of endophytic fungi; they indicated that *Nigrospora oryzae* isolated from *A. sinensis* produced biological activity metabolites such as *β*-phenylethyl alcohol, 11-hydroxycapitulatin B, and Capitulatin B (Li et al. 2014), showing that endophytic *Nigrospora* species from *Aquilaria* play a potential application value in the agarwood-producing industry.

During the survey of endophytic fungi, five strains of *Nigrospora* were isolated and examined from *Aquilaria sinensis* in Guangxi Zhuang Autonomous Region, China, for their morphological characteristics. Phylogenetic relationships based on DNA sequence analyses from nucleotides were investigated to evaluate the taxonomic and phylogenetic status of our strains and other *Nigrospora* species using combined ITS, *TEF1-α*, and *TUB2* sequence data. Full descriptions, illustrations, and phylogenetic analysis results for the two new species and two new host records of *Nigrospora* are provided.

## ﻿Materials and methods

### ﻿Sampling and isolation of endophytic *Nigrospora* species

Healthy leaf samples of *Aquilaria sinensis* were collected in Qinzhou, Guangxi Zhuang Autonomous Region, China. The samples were transported to the laboratory in self-sealing plastic bags stored in insulated ice boxes(Rathnayaka et al. 2025). Leaf samples were rinsed with tap water to remove soil and debris, then aseptically cut into 0.5 cm × 0.5 cm pieces. Surface sterilization was carried out using 75% ethanol for 30 s, followed by 3% H_2_O_2_ for 3 min, and another 30 s treatment with 75% ethanol. The samples were then rinsed three times with sterile distilled water and dried on sterile tissue paper (Du et al. 2022a). After drying, four pieces of leaf tissue were placed on potato dextrose agar (PDA) plates and incubated at room temperature (25 °C). Fungal culture characteristics on PDA were observed after one month. Living cultures were deposited in the Kunming Institute of Botany Culture Collection, Kunming, China (KUNCC).

MycoBank numbers and Facesoffungi numbers were obtained as per the instructions in (https://www.mycobank.org/) and Jayasiri et al. (2015), respectively.

### ﻿DNA extraction, PCR amplification, and sequencing

Fungal mycelia from 30-day-old PDA cultures were scraped off using a sterilized scalpel and transferred into 1.5 mL centrifuge tubes. Genomic DNA was extracted using the Biospin Fungus Genomic DNA Extraction Kit-BSC14S1 (BioFlux®, P.R. China), following the manufacturer’s instructions.

DNA amplification was performed using polymerase chain reaction (PCR) targeting three loci: ITS, *TEF1-α*, and *TUB2*.

The internal transcribed spacer (ITS) region was amplified using the primer pair ITS1 and ITS4 (White et al. 1990). The partial translation elongation factor 1-alpha (*TEF1-α*) region was amplified using EF1-728F and EF2 primers (Carbone and Kohn 1999; O’Donnell et al. 1998). The beta-tubulin (*TUB2*) fragment was amplified using Bt-2a and Bt-2b (Glass and Donaldson 1995). The PCR reaction volume was 25 µL, containing 1 µL forward primer, 1 µL reverse primer, 22 µL JPmix (Tsingke Biotech Co., Ltd.), and 1 µL of template DNA (48 ng/µL). PCR amplification conditions were as follows: initial denaturation at 98 °C for 2 min, followed by 32 cycles of denaturation at 98 °C for 15 s, annealing at 53 °C for 15 s, extension at 72 °C for 45 s, and a final extension at 72 °C for 5 min. Purification and sequencing of PCR products were carried out by Tsingke Biotech Co., Ltd. (Kunming, P.R. China). The DNA product was stored at -20 °C for long-term preservation.

### ﻿Phylogenetic analyses

Phylogenetic analyses were performed following the procedures described in Dissanayake et al. (2020). Sequence quality was assessed using SnapGene v6.1. A BLAST search based on ITS, *TEF1-α*, and *TUB2* sequences was conducted to identify closely related taxa in GenBank (https://www.ncbi.nlm.nih.gov/genbank/), and corresponding sequences were downloaded. *Apiospora obovata* (LC4940) and *Ap. malaysiana* (CBS 102053) were selected as outgroup taxa (Table 1). Sequences were aligned in MEGA v7.0 using MUSCLE, with manual adjustments where necessary (Kumar et al. 2016). Phylogenetic trees were generated using both maximum likelihood (ML) and Bayesian inference (BI) methods via the CIPRES Science Gateway (Miller et al. 2010). The ML analysis was conducted using the GTRGAMMA model with 1000 bootstrap replicates. The BI analysis employed the Invgamma+5 model with 100,000 generations. Resulting trees were visualized in FigTree v1.4.2 (http://tree.bio.ed.ac.uk/software/figtree/), with bootstrap support values indicated at the nodes. The phylogram was rendered in TreeView (Page 1996), illustrated in Microsoft PowerPoint, and exported as a JPEG image using Adobe Photoshop CS5 (Adobe Systems Inc., United States). Newly generated sequences were deposited in GenBank (Table 1). Sequence alignments were submitted to TreeBASE under accession number S31751 (https://treebase.org/).

**Table 1.** Names, culture collection numbers, hosts, and corresponding GenBank accession numbers of the taxa used in the phylogenetic analyses.

Taxa names	Culture collection number	Host	ITS	*TUB2*	*TEF1-α*
*Apiospora obovata*	LC4940	–	KY494696	KY705166	KY705095
*Ap. malaysiana*	CBS 102053	–	NR120273	KF144988	KF145030
*Nigrospora aurantiaca* ^(T)^	CGMCC3.18130 = LC7302	*Nelumbo* sp. (leaf)	KX986064	KY019465	KY019295
*N. aurantiaca*	LC7034	*Musa paradisiaca*	KX986093	KY019598	KY019394
*N. bambusae* ^(T)^	CGMCC3.18327 = LC7114	Bamboo (leaf)	KY385307	KY385319	KY385313
*N. bambusae*	LC7244	Bamboo (leaf)	KY385306	KY385320	KY385314
*N. bambusae*	LC7245	Bamboo (leaf)	KY385305	KY385321	KY385315
*N. camelliae-sinensis*	LC2710	*Castanopsis* sp.	KX985957	KY019484	KY019310
*N. camelliae-sinensis*	LC3287	*Camellia sinensis*	KX985975	KY019502	KY019323
*N. camelliae-sinensis*	LC3496	*Camellia sinensis*	KX985985	KY019510	KY019327
*N. camelliae-sinensis* ^(T)^	CGMCC3.18125 = LC3500	*Camellia sinensis*	KX985986	KY019460	KY019293
*N. camelliae-sinensis*	LC6684	*Camellia sinensis*	KX986046	KY019570	KY019449
***N. camelliae-sinensis***	**KUNCC23-16748**	***Aquilaria sinensis***	**PQ553689**	**PQ613611**	**PQ613606**
*N. chinensis*	LC2696	*Lindera aggregata*	KX985947	KY019474	KY019424
*N. chinensis*	LC3493	*Camellia sinensis*	KX985984	KY019509	KY019434
*N. chinensis*	LC4433	*Castanopsis* sp.	KX986013	KY019536	KY019436
*N. chinensis*	LC4558	Unknown host plant	KX986020	KY019543	KY019441
*N. chinensis* ^(T)^	CGMCC3.18127 = LC4575	*Machilus breviflora*	KX986023	KY019462	KY019422
*N. chinensis*	LC4660	–	–	KY019548	KY019445
*N. chinensis*	LC6631	*Camellia sinensis*	KX986043	KY019569	KY019448
*N. chinensis*	LC6851	Unknown host plant	KX986049	KY019579	KY019450
*N. gorlenkoana* ^(T)^	CBS 480.73	*Vitis vinifera*	KX986048	KY019456	KY019420
*N. gorlenkoana*	JZB3230001	*Cirsium setosum*	MN495939	MN549381	MN54464
***N. guangxiensis***	**KUNCC23-16747**	***Aquilaria sinensis***	**PQ553688**	**PQ613610**	**PQ613605**
*N. guilinensis*	LC7301	*Vitis vinifera*	KX986063	KY019608	KY019404
*N. guilinensis* ^(T)^	CGMCC3.18124 = LC3481	*Nelumbo* sp. (stem)	KX985983	KY019459	KY019292
*N. hainanensis* ^(T)^	CGMCC3.18129 = LC7030	*Musa paradisiaca* (leaf)	KX986091	KY019464	KY019415
*N. hainanensis*	LC6979	*Musa paradisiaca* (leaf)	KX986079	KY019586	KY019416
*N. hainanensis*	LC7031	*Musa paradisiaca*	KX986092	KY019597	KY019417
*N. hainanensis*	LC7042	*Musa paradisiaca* (leaf)	KX986094	KY019599	KY019418
*N. lacticolonia* ^(T)^	CGMCC3.18123 = LC3324	*Camellia sinensis*	KX985978	KY019458	KY019291
*N. lacticolonia*	LC7009	*Musa paradisiaca* (leaf)	KX986087	KY019594	KY019454
*N. musae* ^(T)^	CBS 319.34	*Musa paradisiaca* (fruit)	KX986076	KY019455	KY019419
*N. musae*	LC6385	*Camellia sinensis*	KX986042	KY019567	KY019371
*N. oryzae*	LC6761	*Oryza sativa*	KX986056	KY019574	KY019376
*N. oryzae*	LC7297	*Nelumbo* sp. (leaf)	KX985936	KY019605	KY019400
*N. oryzae*	LC2693	*Neolitsea* sp.	KX985944	KY019471	KY019299
*N. oryzae*	LC2707	*Rhododendron simiarum*	KX985954	KY019481	KY019307
*N. oryzae*	LC4338	*Camellia* sp.	KX986008	KY019532	KY019349
*N. oryzae*	LC4961	*Pittosporum illicioides*	KX986031	KY019553	KY019358
*N. oryzae*	LC5243	Submerged wood	KX986033	KY019555	KY019360
*N. oryzae*	LC6923	*Oryza sativa* L.	KX986051	KY019581	KY019383
*N. oryzae*	JZB3230002	*Phyllostachys nigra*	MN495940	–	MN544639
*N. oryzae*	JZB3230003	*Rudbeckia hirta*	MN495941	–	MN544640
*N. oryzae*	JZB3230004	*Scirpus* sp.	MN495942	MN549382	MN544641
***N. oryzae***	**KUNCC23-16746**	***Aquilaria sinensis***	**PQ553687**	**PQ613609**	**PQ613604**
*N. osmanthi* ^(T)^	CGMCC3.18126 = LC4350	*Osmanthus* sp.	KX986010	KY019461	KY019421
*N. osmanthi*	LC4487	*Hedera nepalensis*	KX986017	KY019540	KY019438
*N. osmanthi*	JZB3230005	*Rosa chinensis*	MN495943	MN549383	MN508179
*N. osmanthi*	JZB3230006	*Rosa chinensis*	MN495944	MN549384	MN508180
*N. osmanthi*	JZB3230007	*Phragmites australis*	MN495945	MN549385	MN508181
*N. osmanthi*	JZB3230008	*Cirsium setosum*	MN495946	MN549386	MN508182
*N. osmanthi*	JZB3230009	*Phyllostachys nigra*	MN495947	MN549387	MN508183
*N. osmanthi*	JZB3230010	*Phyllostachys nigra*	MN495948	MN549388	MN508184
*N. osmanthi*	JZB3230011	*Rudbeckia hirta*	MN495949	MN549389	MN508185
***N. pubeiensis***	**KUNCC23-16745**	***Aquilaria sinensis***	**PQ553686**	**PQ613608**	**PQ613603**
*N. pyriformis* ^(T)^	CGMCC3.18122 = LC2045	*Citrus sinensis*	KX985940	KY019457	KY019290
*N. pyriformis*	LC2688	*Lindera aggregata*	KX985941	KY019468	KY019297
*N. pyriformis*	LC2694	*Rubus reflexus*	KX985945	KY019472	KY019300
*N. pyriformis*	LC3099	*Camellia sinensis*	KX985971	KY019498	KY019322
*N. pyriformis*	LC3292	*Camellia sinensis*	KX985976	KY019503	KY019324
*N. rubi* ^(T)^	CGMCC3.18326 = LC2698	*Rubus* sp.	KX985948	KY019475	KY019302
*N. rubi*	JZB3230012	*Fraxinus* sp.	MN495950	–	MN54464
*N. sphaerica*	LC7312	*Nelumbo* sp. (leaf)	KX985935	KY019618	KY019414
*N. sphaerica*	LC7298	*Nelumbo* sp. (leaf)	KX985937	KY019606	KY019401
*N. sphaerica*	LC2840	*Harpullia longipetala*	KX985965	KY019492	KY019318
*N. sphaerica*	LC3477	*Camellia sinensis*	KX985982	KY019508	KY019326
*N. sphaerica*	LC4264	*Rhododendron arboretum*	KX985993	KY019517	KY019334
*N. sphaerica*	LC4307	*Rhododendron arboretum*	KX986005	KY019529	KY019346
*N. sphaerica*	LC5901	Submerged wood	KX986034	KY019556	KY019361
*N. sphaerica*	LC6294	*Camellia sinensis*	KX986044	KY019565	KY019369
*N. sphaerica*	LC6996	*Musa paradisiaca* (leaf)	KX986085	KY019592	KY019390
*N. sphaerica*	JZB3230013	*Cirsium setosum*	MN495951	MN549390	MN544642
*N. sphaerica*	JZB3230014	*Phragmites australis*	MN495952	MN549391	MN544643
*N. sphaerica*	JZB3230015	*Fraxinus* sp.	MN495953	MN549392	MN544644
*Nigrospora* sp. 1	LC2725	*Symplocos zizyphoides*	KX985960	KY019487	KY019313
*Nigrospora* sp. 1	LC4566	*Lithocarpus* sp.	KX986022	KY019545	KY019354
*Nigrospora* sp. 2	LC6704	*Camellia sinensis*	KX986047	KY019571	KY019373
*N. vesicularis*	LC0322	Unknown	KX985939	KY019467	KY019296
*N. vesicularis* ^(T)^	CGMCC3.18128 = LC7010	*Musa paradisiaca* (leaf)	KX986088	KY019463	KY019294
*N. zimmermanii*	CBS 167.26	Unknown	KY385308	KY385318	KY385312
*N. zimmermanii* ^(T)^	CBS 290.62	*Saccharum officinarum* (leaf)	KY385309	KY385317	KY385311
*N. zimmermanii*	CBS 984.69	*Saccharum officinarum* (leaf)	KY385310	KY385322	KY385316
*Nigrospora* sp.	YL-2024a	–	OP677969	PP103614	PP103590
*N. shadeganensis*	SCUA-Saf-N27	–	PP256499	PP263821	PP263812
*N. coryli*	W18	*Corylus heterophylla*	PP218065	PP218065	PP461302
*N. humicola*	CFCC 56884	Soil	ON555686	ON557392	ON557394
*N. yunnanensis*	GUCC24-0008	–	PP915796	PP947937	PP947933

– Refers to the data unavailability, ^(T)^refers to the type specimens, and the taxa produced in this study are in bold.

## ﻿Results

### ﻿Phylogenetic analyses

The combined ITS, *TEF1-α*, and *TUB2* sequence dataset comprised 77 taxa, with *Apiospora obovata* (LC4940) and *Ap. malaysiana* (CBS 102053) selected as outgroup taxa.

**Figure 1.**
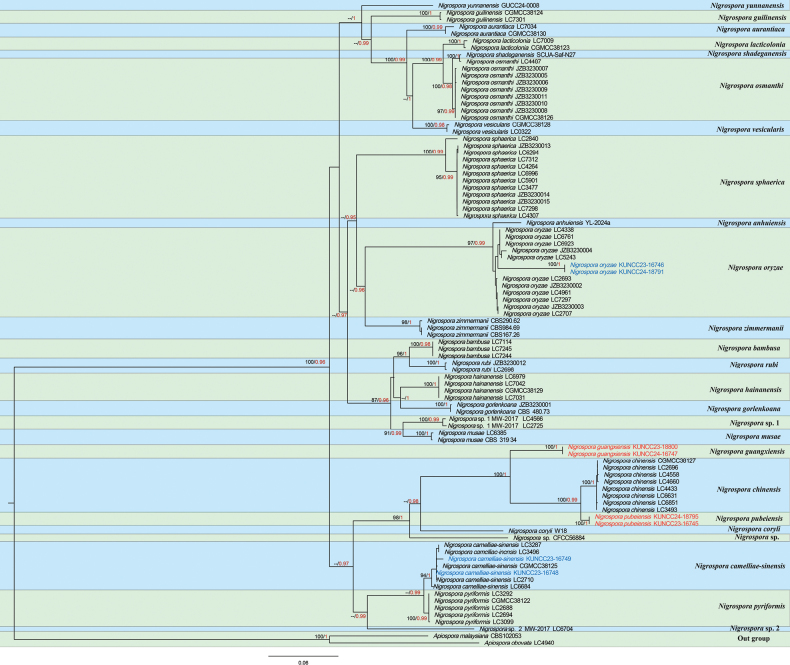
Tree generated by RAxML analysis showing the placement of *Nigrospora* species based on combined ITS, *TEF1-α*, and *TUB2* sequence data. Bootstrap support values for ML (left) ≥ 75% and Bayesian posterior probabilities (PP, right) ≥ 0.9 are indicated at the nodes. The tree is rooted with *Ap. obovata* (LC4940) and *Ap. malaysiana* (CBS 102053). Strains generated in this study are shown in red and blue bold; red indicates new species, and blue indicates new host records.

Bayesian inference (BI) and maximum likelihood (ML) analyses of the combined dataset were conducted to determine the placement of our new taxa, infer relationships at the intrageneric level, and resolve phylogenetic relationships within Apiosporaceae. The phylogenetic trees derived from BI and ML analyses exhibited largely similar topologies and were consistent with previous studies based on ML analysis (Li et al. 2017; Su et al. 2018). The best-scoring RAxML tree is shown in Fig. 1, with a final ML optimization likelihood value of –10322.409322 (ln). The dataset consisted of 1,595 total characters, including gaps (ITS: 1–591 bp; *TEF1-α*: 592–1170 bp; *TUB2*: 1171–1595 bp). RAxML analysis yielded 1,595 distinct alignment patterns, with 33.97% of characters undetermined or containing gaps.

#### ﻿ Nigrospora camelliae-sinensis

Taxon classificationFungiAmphisphaerialesApiosporaceae

Mei Wang & L. Cai, in Wang, Liu, Crous & Cai, Persoonia 39: 127 (2017)

BACB0194-7817-50D4-A2D7-8E81E28862B4

820731

Fig. 2

##### Description.

***Endophytic*** from healthy leaves of *Aquilaria sinensis*. Sexual morph: Undetermined. Asexual morph: ***Hyphae*** branched, septate, hyaline, or light grey, 2–4 µm diam. ***Conidiophores*** 3–6 µm diam. micronematous, mononematous, solitary, smooth, branched or not, 0–1 septate, hyaline to subhyaline, consisting of 1–2 cells or usually reduced to conidiogenous cells. ***Conidiogenous cells*** 5.5–7.2 × 6.3–9.4 µm diam. x̄ = 5.8 × 12.1 µm, n = 30), monoblastic, discrete, determinate, smooth, subglobose to ampulliform, subhyaline to light brown. ***Conidia*** 8–13 µm diam. x̄ = 11 µm, n = 30) solitary, smooth-walled, aseptate, spherical, light grey to black, globose or subglobose.

##### Cultural characteristics.

Mycelium effusing on PDA within 12 hours from the edges of the surface sterilized leaf tissue piece. Colonies growing on PDA, hairy, black, reaching 9 cm in 7 days at 30 °C; mycelium partly superficial, partly immersed, slightly effuse, radially striate, with an irregular edge, black. Asexual conidia spores were formed after 25 days on PDA. Sexual spores were not formed within 60 days.

**Figure 2.**
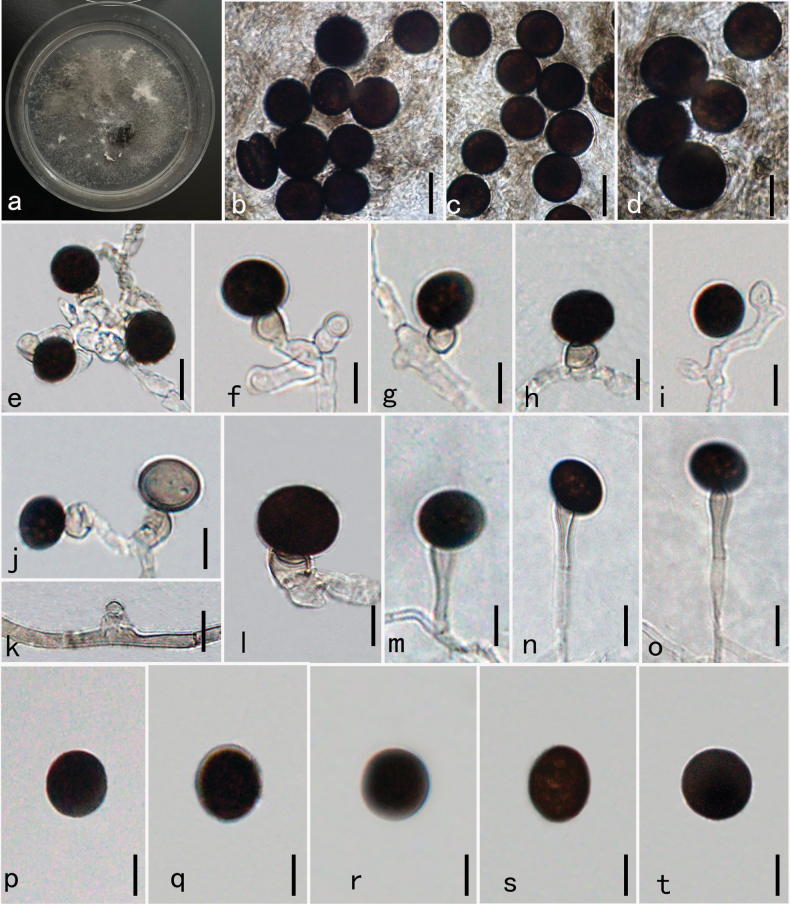
*Nigrospora camelliae-sinensis* (HKAS–134953, new host record). **a.** Culture on PDA for 30 days; **b–d.** Mass of conidia; **e–j.** Conidiogenous cells attached conidia; **k.** Conidiogenous cell arising from hyphae; **l–o.** Conidiophores bearing conidia; **p–t.** Conidia. Scale bars: 10 μm (**b–t**).

##### Material examined.

**China** • Guangxi Zhuang Autonomous Region, Pubei City, in healthy living leaves of *Aquilaria sinensis*, September 25, 2020, Shiyu Zhang, GX9-3 (HKAS 134953, new host record) . Living culture KUNCC23-16748.

##### Known distributions

**(based on molecular data)**: China (Wang et al. 2017, this study).

##### Known hosts

**(based on molecular data)**: *Aquilaria sinensis* (this study), *Camellia sinensis* (Wang et al. 2017), *Castanopsis* sp. (Wang et al. 2017), *Musa paradisiaca* (Wang et al. 2017).

##### Notes.

*Nigrospora camelliae-sinensis* has been reported to have a cosmopolitan distribution and a broad host range (Wang et al. 2017). In this study, our isolation (KUNCC23-16748), collected from *Aquilaria sinensis* in Guangxi Zhuang Autonomous Region, China, clustered in one single clade with *N. camelliae-sinensis* (94% ML, 1PP). Its morphological characteristics were in good agreement with those of *N. camelliae-sinensis.* Therefore, we regard this isolation (KUNCC23-16748) as *N. camelliae-sinensis* collected from *Aquilaria sinensis* for the first time. The study showed that *N. camelliae-sinensis* is also characterized by having determinate and micro- and mononematous, subcylindrical conidiophores.

#### ﻿ Nigrospora guangxiensis

Taxon classificationFungiAmphisphaerialesApiosporaceae

S.Y. Zhang, J.F. Li & K.D. Hyde sp. nov.

38F8999F-AAAB-59E3-8093-81A7A5415CC2

859462

Facesoffungi Number: FoF17621

Fig. 3

##### Etymology.

Named after the type location, Guangxi Zhuang Autonomous Region, China.

##### Holotype.

HKAS 134952.

##### Description.

***Endophytic*** from healthy leaves of *Aquilaria sinensis*. Sexual morph: Undetermined. Asexual morph: ***Hyphae*** branched, septate, hyaline to pale brown, 1–3 µm diam. ***Conidiophores*** usually reduced to conidiogenous cells, which are dispersed on hyphae. ***Conidiogenous cells*** 4.5–6 × 5.3–7.3 µm diam. x̄ = 5 × 6.8 µm, n = 30), discrete, solitary, monoblastic, determinate, subglobose, pale brown to brown colored. *Conidia* 8–14 μm diam. x̄ = 10.5 µm, n = 30), solitary, discrete, dark brown to black, globose or subglobose.

##### Cultural characteristics.

Mycelium effusing on PDA within 12 hours from the edges of the surface sterilized leaf tissue piece. Colonies growing on PDA, hairy, reaching 9 cm in 7 days at 30 °C; mycelium partly superficial, partly immersed, slightly effuse, radially striate, with irregular edge, initially greyish-brown, becoming black colored with age; asexual spores were formed after 25 days on PDA, and sexual spores not formed within 60 days on PDA.

**Figure 3.**
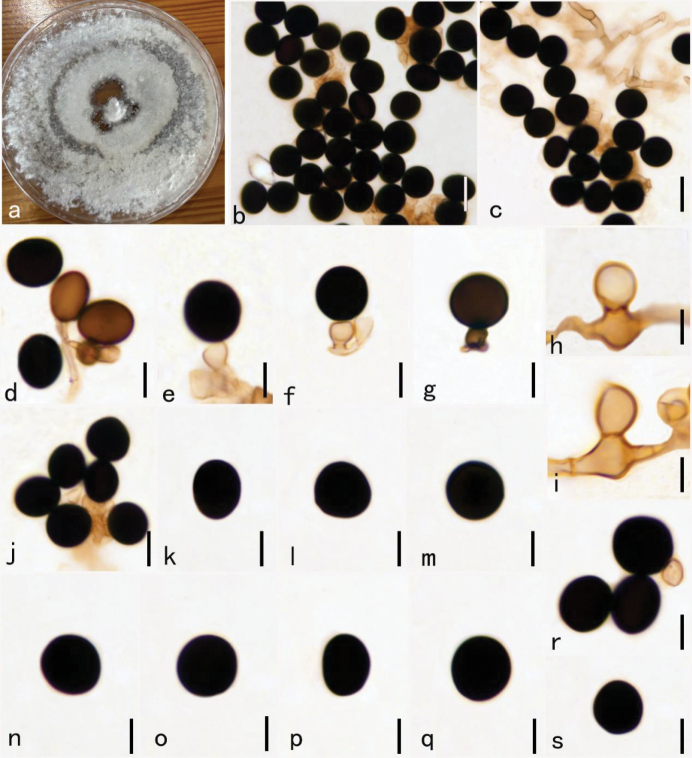
*Nigrospora guangxiensis* (HKAS–134952, holotype). **a.** Culture on PDA for 30 days; **b, c.** Mass of conidia; **d–g.** Conidiogenous cells attached conidia; **h, i.** Conidiophores; **j–s.** Conidia. Scale bars: 10 μm (**b–s**).

##### Known distribution

**(based on molecular data)**: China (this study).

##### Known hosts.

*Aquilaria sinensis* (this study).

##### Material examined.

**China** • Guangxi Zhuang Autonomous Region, Pubei city, on healthy living leaves of *Aquilaria sinensis*, September 25, 2020, Shiyu Zhang, GX4-1 (HKAS 134952, holotype) ; extype living culture, KUNCC23-16747.

##### Notes.

In the multi-locus phylogenetic analyses, our strain *Nigrospora guangxiensis* (KUNCC23-16747) formed a separate branch basal (97% ML, 1PP) to *N. pubeiensis* (KUNCC23-16745) and *N. chinensis* (eight strains). *Nigrospora guangxiensis* differs from *N. pubeiensis* in having smaller conidiogenous cells (5 × 6.8 µm *vs.* 5.8 × 9.4 µm) and shorter conidia (10.5 µm vs. 11.3 µm). Additionally, its initial culture on PDA medium is greyish-brown. *Nigrospora guangxiensis* is distinct from *N. chinensis* in having smaller conidia (10.5 µm vs. 12.97 ± 1.07 µm), pale brown to brown-colored conidiogenous cells, and sterile cells, which are absent in *N. guangxiensis* (Wang et al. 2017). A nucleotide base comparison of these species is shown in the notes of *N. pubeiensis* and Table 2.

**Table 2.** Base pair comparison in ITS, *TEF1-α*, and *TUB2* sequences of *N. chinensis*, *N. pubeiensis*, and *N. guangxiensis*.

Species	Base pair comparison in ITS, *TUB2*, and *TEF1-α*		
	ITS	*TUB2*	*TEF1-α*
*N. chinensis* (CGMCC-38127) *vs. N. pubeiensis* (KUNCC23-16745)	3/492(0.6%)	26/365(7.1%)	0
*N. chinensis* (CGMCC-38127) *vs. N. guangxiensis* (KUNCC23-16747)	10/492(2.0%)	26/373(7.0%)	62/425(14.5%)
*N. guangxiensis* (KUNCC23-16747) *vs. N. pubeiensis* (KUNCC23-16745)	9/511(1.8%)	0	62/425(14.5%)

#### ﻿ Nigrospora oryzae

Taxon classificationFungiAmphisphaerialesApiosporaceae

(Berk. & Broome) Petch, J. Indian bot. Soc. 4: 24 (1924)

CB306075-7C85-5702-9640-4036E33016A2

253729

Facesoffungi Number: FoF06596

Fig. 4

Monotospora oryzae Berk. & Broome, J. Linn. Soc., Bot. 14: 99. 1873. Basionym.
Khuskia oryzae H.J. Huds., Trans. Brit. Mycol. Soc. 46: 358. 1963. Synonym.

##### Description.

***Endophytic*** from healthy leaves of *Aquilaria sinensis*. Sexual state: Undetermined. Asexual state: Hyphomycetes. ***Hyphae*** 1–3.5 µm diam. branched, smooth, septate, hyaline, or subhyaline. ***Conidiophores*** 2–5 µm diam. micronematous, solitary in sporodochia, subcylindrical, pale grey, smooth, 0–2-septate, branched or not, usually reduced to conidiogenous cells. ***Conidiogenous cells*** 4.7–5.2 × 6.3–7.4 µm diam. x̄ = 5.0 × 6.9 µm, n = 50), monoblastic, solitary, determined, hyaline to subhyaline, ampliform to subspherical, discrete, smooth-walled. ***Conidia*** 8–13.5 µm diam x̄ = 11.3 µm, n = 50) solitary, globose or subglobose, black, shiny, smooth, aseptate.

##### Cultural characteristics.

Mycelium effusing on PDA within 12 hours from the edges of the surface-sterilized leaf tissue piece. Colonies growing on PDA, hairy, black, reaching 5 cm in 7 days at 30 °C; mycelium partly superficial, partly immersed, slightly effuse, radially striate, with an irregular edge, dark brown to black colored; asexual spores were formed after 25 days on PDA, and sexual spores were not formed within 60 days on PDA.

**Figure 4.**
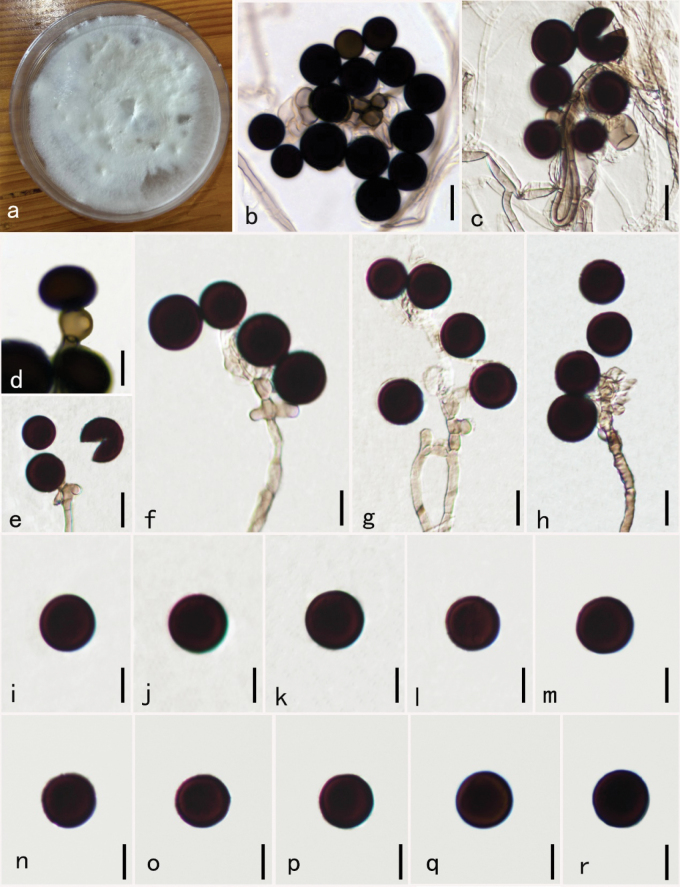
*Nigrospora oryzae* (HKAS—134951), new host and geographical record. **a.** Culture on PDA for 30 days; **b, c.** Mass of conidia; **d, e.** Conidiogenous cells attached conidia; **f–h.** Conidiophores bearing conidia; **i–r.** Conidia. Scale bars: 10 μm (**a–r**).

##### Material examined.

**China** • Guangxi Zhuang Autonomous Region, Pubei city, on healthy living leaves of *Aquilaria sinensis*, September 25, 2020, Shiyu Zhang, GX2-3 (HKAS 134951, new host record) . Living culture KUNCC23-16746.

##### Known distribution

**(based on molecular data)**: Australia (Barkat et al. 2016), Bangladesh (Begum et al. 2018), China (Sun et al. 2011; Chen and Kirschner2018; He et al. 2019; Zhang et al. 2019, this study), Iran (Hashemian et al. 2014), Italy (Lorenzini et al. 2016), Kazakhstan (Eken et al. 2016), Pakistan (Alam et al. 2017), Sri Lanka (Wang et al. 2017), and Thailand (Hyde et al. 2020).

##### Known hosts

**(based on molecular data)**: *Acer truncatum* (Sun et al. 2011), *Actinidia* sp. (Li et al. 2018), *Aloe vera* (Alam et al. 2017), *Aquilaria sinensis* (This study), *Castanopsis* sp. (Wang et al. 2017), *Cephalotaxus sinensis* (Wang et al. 2017), *Citrullus lanatus* (Chen and Kirschner 2018), *Citrus reticulata* (Wang et al. 2017), *Cleyera japonica* (Wang et al. 2017), *Daphniphyllum macropodum* (Wang et al. 2017), *Daphniphyllum oldhamii* (Wang et al. 2017), *Gossypium hirsutum* (Zhang et al. 2019), *Hamamelis mollis* (Wang et al. 2017), *Nelumbo nucifera* (Chen and Kirschner 2018), *Nelumbo* sp. (Wang et al. 2017), *Neolitsea* sp. (Wang et al. 2017), *Oryza sativa* (Wang et al. 2017), *Osmanthus fragrans* (Wang et al. 2017), *Osmanthus* sp. (Wang et al. 2017), *Pennisetum americanum* (Hashemian et al. 2014), *Pentactina rupicola* (Wang et al. 2017), *Photinia serrulata* (He et al. 2019), *Phyllostachys heterocycla* (Hyde et al. 2020), *Rhododendron simiarum* (Wang et al. 2017), *Rhododendron* sp. (Wang et al. 2017), *Rubus reflexus* (Wang et al. 2017), *Rubus* sp. (Wang et al. 2017), *Symplocos zizyphoides* (Wang et al. 2017), *Ternstroemia* sp. (Wang et al. 2017), *Triticum aestivum* (Eken et al. 2016; Barkat et al. 2016), *Tutcheria microcarpa* (Wang et al. 2017), *Vaccinium corymbosum* (Zhang et al. 2019), *Vitis vinifera* (Lorenzini et al. 2016).

##### Notes.

*Nigrospora oryzae* has been reported to have a cosmopolitan distribution and a broad host range (Wang et al. 2017). In this study, our isolate (KUNCC23-16746) clustered in the same clade (100% ML, 1 PP) as *N. oryzae*, and its morphological characteristics were consistent with those of *N. oryzae* (Wang et al. 2017). Therefore, we regard this isolate (KUNCC23-16746) as *N. oryzae*, which was collected from *Aquilaria sinensis* in the Guangxi Zhuang Autonomous Region, China, for the first time.

#### ﻿ Nigrospora pubeiensis

Taxon classificationFungiAmphisphaerialesApiosporaceae

S.Y. Zhang, J.F. Li & K.D. Hyde sp. nov.

3676BF35-A7EA-5015-A981-698FE3D645F7

859460

Facesoffungi Number: FoF17622

Fig. 5

##### Etymology.

Named after the location from which it was collected, Pubei, Guangxi Zhuang Autonomous Region, China.

##### Holotype.

HKAS 134950.

##### Description.

***Endophytic*** from healthy leaves of *Aquilaria sinensis*. Sexual morph: Undetermined. ***Asexual morph*: *Hyphae*** 3–6 µm diam. branched, smooth, septate, hyaline, or subhyaline. ***Conidiophores*** 1–4 µm diam, micronematous, solitary, subcylindrical, hyaline to pale grey, smooth, 0–2-septate, branched or not, usually reduced to conidiogenous cells. ***Conidiogenous cells*** 5–6.3 × 6.3–11.3 µm diam. (x̄ = 5.8 × 9.4 µm, n = 20), discrete, solitary, monoblastic, determinate, subglobose, straight, smooth, hyaline or dark brown. ***Conidia*** 6–14 μm diam (x̄ = 11.3 µm, n = 20), solitary, granular, dark brown to black, globose, or subglobose.

##### Cultural characteristics.

Mycelium effusing on PDA within 12 hours from the edges of the surface-sterilized leaf tissue piece. Colonies growing on PDA, hairy, black, reaching 9 cm in 7 days at 30 °C; mycelium partly superficial, partly immersed, slightly effuse, radially striate, with irregular edge, initially grayish white, becoming black colored with age; asexual spores were formed after 25 days on PDA, and sexual spores not formed within 60 days on PDA.

##### Known distribution

**(based on molecular data)**: China (this study).

##### Known hosts.

*Aquilaria sinensis* (this study).

##### Material examined.

**China** • Guangxi Zhuang Autonomous Region, Pubei City, in healthy living leaves of *Aquilaria sinensis*, September 25, 2020, Shiyu Zhang, GX2-2 (HKAS 134950, ***holotype*)**. Ex-type living culture at KUNCC23-16745.

##### Notes.

*Nigrospora pubeiensis* is described herein as a new species, which resembles other species in the genus in having granular, dark brown to black, globose, or subglobose conidia, but differs in its short conidiophores, sometimes reduced to conidiogenous cells. The strain representing *N. pubeiensis* (KUNCC23-16745) clustered in a supported clade (100% ML, 1 PP) and is closely related to *N. guangxiensis* (KUNCC23-16747) and *N. chinensis* (CGMCC-38127). However, *N. pubeiensis* is distinct in nucleotide base pair comparison with *N. guangxiensis* (KUNCC23-16747) across the ITS gene region (9/511 bp, 1.8% difference, no gap) and the *TEF1-α* gene region (62/425 bp, 14.5% difference, no gap), *TUB2* (0); it differs with *N. chinensis* (CGMCC-38127) across the ITS gene region (3/492 bp, 0.6% difference, no gap) and the *TUB2* gene region (26/365 bp, 7.1% difference, no gap), *TEF1-α* (0). They also have different conidiophore structures. Compared with the photoplate from Wang et al. (2017), the conidiophore of *N. chinensis* has an ampulliform structure, but *N. pubeiensis* does not. Based on distinct morphological characteristics and phylogenetic support, we regard this isolation (KUNCC23-16745) as a new species, *N. pubeiensis*, which was collected from *Aquilaria sinensis* in Guangxi Province, China.

**Figure 5.**
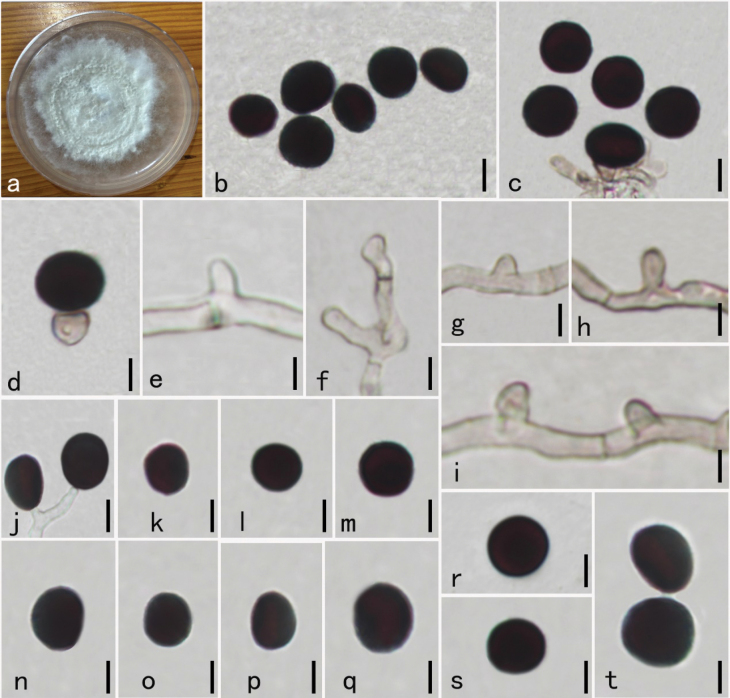
*Nigrospora pubeiensis* (HKAS–134950, holotype). **a.** Culture on PDA for 30 days; **b.** Mass of conidia; **c, d.** Conidiogenous cells attached conidia; **e–i.** Conidiophores; **j.** Conidiophores bearing conidia; **k–t.** Conidia. Scale bars: 10 μm (**b–t**).

## ﻿Discussion

The discovery of two novel *Nigrospora* species (*N. pubeiensis* and *N. guangxiensis*), along with new host records for *N. oryzae* and *N. camelliae-sinensis*, highlights the adaptability of *Nigrospora* to diverse ecological niches. The broad host range of *Nigrospora* on *Aquilaria* spp. may be linked to its ability to exploit host-specific chemical environments. For instance, endophytic fungi in *A. sinensis* often interact with host-derived secondary metabolites, such as sesquiterpenes and chromones, which are abundant in agarwood resin (Li et al. 2025). These compounds may create selective pressures that favor fungal lineages capable of tolerating or metabolizing them (Li et al. 2025). Evidence from other studies shows that *Nigrospora* species produce bioactive metabolites (e.g., antimicrobial compounds), which could confer competitive advantages in colonizing new hosts or resisting host defenses (Metwaly et al. 2014; Kuang et al. 2022).

Ecologically, the occurrence of *Nigrospora* on *A. sinensis* aligns with observations that endophytic fungal communities shift dynamically during agarwood formation. For example, fungal diversity decreases as resinous compounds accumulate, suggesting a trade-off between host defense and fungal survival (Li et al. 2025). Similar patterns have been observed in *Fusarium*-induced agarwood, where dominant fungi such as *Colletotrichum* thrive in resin-rich tissues (Fang et al. 2024). This implies that *Nigrospora* may occupy niches where host metabolites are less inhibitory or where their secondary metabolites counteract host defenses.

Evolutionarily, the divergence of *Nigrospora* lineages may reflect co-adaptation with *Aquilaria* hosts. Multi-gene phylogenies (e.g., ITS, *TEF1*, and *TUB2*) reveal that fungal speciation often correlates with host specificity (Tibpromma et al. 2021). For instance, *Nemania* species isolated from *A. sinensis* produce sesquiterpenoids identical to those found in agarwood, suggesting a symbiotic relationship in which the fungi contribute to resin biosynthesis (Tibpromma et al. 2021). Analogously, *Nigrospora* may have evolved metabolic pathways that enable coexistence with *Aquilaria*’s chemical defenses, thus facilitating colonization across diverse hosts.

## Supplementary Material

XML Treatment for Nigrospora camelliae-sinensis

XML Treatment for Nigrospora guangxiensis

XML Treatment for Nigrospora oryzae

XML Treatment for Nigrospora pubeiensis

## ﻿Additional information

### Use of AI

No use of AI was reported.

### Author ORCIDs

Shiyu Zhang https://orcid.org/0009-0001-4174-6103

Hongbo Jiang https://orcid.org/0000-0001-9655-4662

Kevin D. Hyde https://orcid.org/0000-0002-2191-0762

Prapassorn Damrongkool Eungwanichayapant https://orcid.org/0000-0001-8005-4137

### Data availability

All of the data that support the findings of this study are available in the main text.
